# Antitumoral and antimetastatic activity of Maitake D-Fraction in triple-negative breast cancer cells

**DOI:** 10.18632/oncotarget.25174

**Published:** 2018-05-04

**Authors:** Eliana Noelia Alonso, María Julia Ferronato, María Eugenia Fermento, Norberto Ariel Gandini, Alejandro López Romero, Josefina Alejandra Guevara, María Marta Facchinetti, Alejandro Carlos Curino

**Affiliations:** ^1^ Laboratorio de Biología del Cáncer, Instituto de Investigaciones Bioquímicas de Bahía Blanca (INIBIBB), Universidad Nacional del Sur (UNS)–CONICET, Departamento de Biología, Bioquímica y Farmacia (UNS), Bahía Blanca, Argentina; ^2^ Departamento de Hematología, IACA Laboratorios, Bahía Blanca, Argentina

**Keywords:** Maitake D-Fraction, mushroom, triple-negative breast cancer, antitumoral, antimetastatic

## Abstract

Triple-negative breast cancer (TNBC) is associated with poor prognosis, high local recurrence rate and high rate of metastasis compared with other breast cancer subtypes. In addition, TNBC lacks a targeted therapy. This scenario highlights the need for novel compounds with high potential for TNBC treatment. In this regard, natural products are important sources of anticancer drugs. D-Fraction, a proteoglucan extracted from the edible and medicinal mushroom *Grifola frondosa* (Maitake), is a dietary supplement that has been shown to exert both immunostimulatory and immune-independent antitumoral effects on some cancer types. However, its antitumoral potential in TNBC is unknown. Therefore, we employed TNBC cells to investigate if D-Fraction is able to attenuate their aggressive phenotype. We found that D-Fraction decreases MDA-MB-231 cell *viability* through *apoptosis* induction and reduces their metastatic potential. D-Fraction increases *cell-cell adhesion* by increasing E-cadherin protein levels and β-catenin membrane localization, and increases *cell-substrate adhesion*. D-Fraction also decreases cell *motility* by affecting actin cytoskeleton rearrangements, and *proteolytic activity* of MMP-2 and MMP-9. Furthermore, D-Fraction decreases the *invasive* capacity of MDA-MB-231 cells. In concordance, D-Fraction *retards tumor growth* and *reduces lung metastases* in a xenograft model. Altogether, these results suggest the potential therapeutic role of D-Fraction in aggressive TNBC.

## INTRODUCTION

Despite advances in the diagnosis and treatment of human malignancy, cancer remains among the leading causes of morbidity and mortality worldwide [1], with 8.2 million deaths attributed to cancer in 2012 [2]. Breast cancer is, by far, the most frequently diagnosed malignant neoplasm and a leading cause of cancer death in females worldwide [3], accounting for 25.1% of cancer diagnoses (1.67 million women) and 14.7% of cancer deaths (521,907 women) in 2012 [2]. Approximately 10–20% of all mammary tumors are TNBC.

TNBC is defined immunohistochemically as a mammary carcinoma that lacks expression of estrogen receptor (ER), progesterone receptor (PR) and human epidermal growth factor receptor 2 (HER2) [4]. This tumor phenotype is associated with high proliferative rate, early and high local recurrence rate [5, 6], high rate of metastasis compared with other breast cancer subtypes [6] and poor survival rates [5, 6]. In addition to these aggressive characteristics, TNBC lack a targeted therapy [7]. Their lack of expression of ER, PR and HER2 makes them insensitive to endocrine and HER2-based therapies. Hence, TNBC is currently treated with standard cytotoxic chemotherapy [8]. In this context, there is a clear need for novel compounds with low toxicity and high potential for treatment or chemoprevention of TNBC.

Mushrooms have attracted the interest of many food and biopharmaceutical areas given their well known nutritional and medicinal values. In cancer research, natural products have played a very important role in the drug discovery and development process [9] and particularly, many mushroom species are considered small pharmaceutical factories producing hundreds of compounds with biological activity [10]. *Grifola frondosa*, commonly known as Maitake, is an edible and medicinal mushroom that has been used for centuries by the oriental medicine, especially in countries like China, India, Japan and Korea [11]. D-Fraction is a standardized form of protein-bound β-glucans (proteoglucan) extracted from the fruit bodies of Maitake, considered as a dietary supplement. While most mushroom-derived β-glucans have a 1,3 main chain with 1,6 branches only, β-glucans found in D-Fraction have a unique and complex structure, containing both a 1,6 main chain having a greater degree of 1,3 branches, and a 1,3 main chain having 1,6 branches [12, 13]. Animal studies and early clinical trials showed that ingestion of Maitake D-Fraction is safe with no toxic or adverse effects and that may even provide health benefits and improvements in the treatment of some types of cancer [12, 14].

The antitumoral activity of D-Fraction has been the focus of multiple investigations. Initially this effect was attributed exclusively to their immunomodulatory capacity. Using different animal models, it was found that D-Fraction not only activates effectors cells of both the innate [14–20] and adaptive [18, 21] immune system but also potentiates the production and release of lymphokines and interleukins that amplify immune responses. More recently, increasing evidence shows that D-Fraction also exhibits immune-independent antitumoral effects on some types of cancer. Thus, the proteoglucan has antiproliferative or cytotoxic effect on PC3 (prostate cancer) [22], T24 (bladder cancer), HepG2 (liver cancer), U89 (brain cancer), HL60 (leukemia) [23], MCF7 and LM3 (breast cancer) [23–25], ACHN cells (kidney cancer) [26, 27] and three canine cancer cell lines, but it does not affect the viability of A549 (lung cancer) and AGS cells (gastric cancer) [28].

Particularly with regard to breast cancer, we have demonstrated that Maitake D-Fraction decreases the viability of hormone-dependent MCF7 cells [24] and modulates the expression of genes associated with cell proliferation, cell death, migration, invasion and metastasis among others [29]. Moreover, considering that breast cancer is a heterogeneous disease constituted by a broad spectrum of tumor subtypes, we demonstrated that D-Fraction decreases the viability and metastatic potential of hormone-independent LM3 cells *in culture* and reduces the tumor burden and the number of lung metastases in the LM3 syngeneic murine model [25]. However, it remains unknown whether Maitake D-Fraction has antitumoral effects in TNBC, an aggressive tumor subtype that have a limited number of treatment choices.

MDA-MB-231 is a human, highly metastatic, TNBC cell line that has been widely used as cell model to study TNBC development and progression and to investigate new drugs against TNBC. Therefore, in the present study we employed MDA-MB-231 cells to investigate the effect of Maitake D-Fraction on the cellular processes that are frequently deregulated in tumor cells and linked to development and malignancy of cancer. In addition, another TNBC cell line, the murine 4T1, was used to evaluate whether the effects of D-Fraction are cell line-independent. These results will give information regarding the potential therapeutic use of D-Fraction in TNBC.

## RESULTS

### Maitake D-Fraction decreases the viability of TNBC MDA-MB-231 and 4T1 cells through apoptosis induction

**Figure 1:**
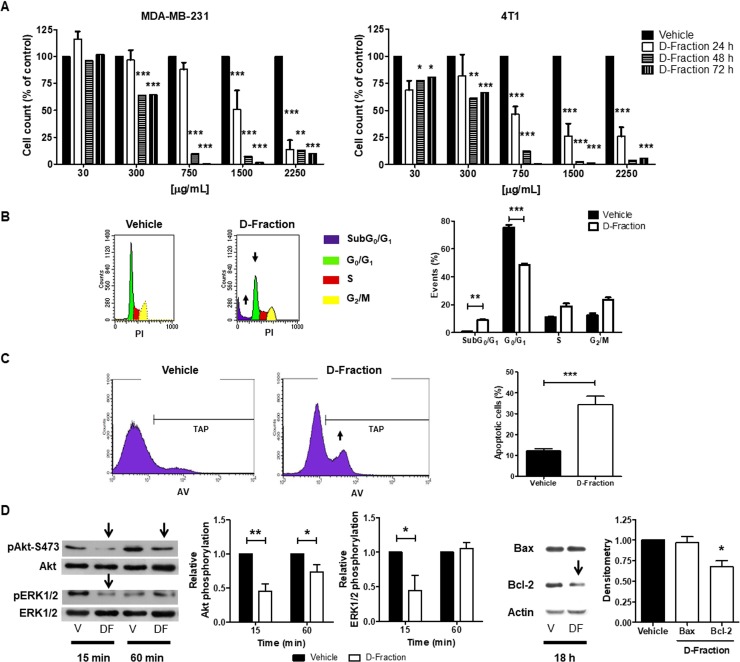
Maitake D-Fraction decreases the viability of TNBC MDA-MB-231 and 4T1 cells through apoptosis induction (**A**) Cell count was assessed in MDA-MB-231 and 4T1 cells after 24, 48 or 72 h of treatment with different concentrations of D-Fraction or vehicle. Data show the percentage of cells in relation to vehicle-treated cells. The bars represent the mean ± SEM of at least two independent experiments. (**B**) Cell cycle analysis of MDA-MB-231 cells after treatment with D-Fraction (IC_50_, 24 h) or vehicle. The increase in subG_0_/G_1_ and the decrease in G_0_/G_1_ cell populations are indicated by arrows and plotted in a graph. Mean ± SD of one representative experiment. Two-way ANOVA and Bonferroni post tests. (**C**) Apoptosis analysis of MDA-MB-231 cells after treatment with D-Fraction (IC_50_, 24 h) or vehicle. The increase in the total apoptotic population (TAP) after D-Fraction treatment is indicated by an arrow and plotted in a graph. Mean ± SD of one representative experiment. Student´s *t* test. (**D**) WB analysis for pAkt-S473, Akt, pERK1/2, ERK1/2, Bax and Bcl-2 proteins in MDA-MB-231 cells after treatment with D-Fraction (IC_50_, 15 min, 60 min and 18 h) or vehicle. Representative blots of three independent experiments are shown. The densitometry mean ± SD is depicted. Two-way ANOVA and Bonferroni post tests, and Student´s *t* test were performed. ^*^p < 0.05, ^**^p < 0.01, ^***^p < 0.001.

To begin to investigate the antitumor effects of D-Fraction in TNBC, we first examined its effects on MDA-MB-231 and 4T1 cell viability. For this purpose, the cells were treated with different concentrations of D-Fraction (30, 300, 750, 1500 and 2250 μg/mL) and for different incubation times (24, 48 and 72 h). Then, manual cell count and WST-1 assay were performed. As shown in Figure 1A, a decrease in the cell count of both TNBC cell lines was observed, being this effect dose- and time-dependent. The IC_50_ values of D-Fraction for MDA-MB-231 cells at 24, 48 and 72 h were 1050 μg/mL, 322.2 μg/mL and 238.2 μg/mL, respectively. Similar IC_50_ values for 4T1 cells were found, being 1077.7 μg/mL, 352 μg/mL and 314.5 μg/mL at 24, 48 and 72 h respectively. Therefore, for the subsequent studies we chose the IC_50_ at 24 h of each cell line (1050 μg/mL for MDA-MB-231 cells and 1077.7 μg/mL for 4T1 cells). The colorimetric WST-1 assay confirmed the decrease of cell viability induced by D-Fraction in the TNBC cell lines (data not shown).

In order to determine whether D-Fraction exerts a cytostatic or apoptotic effect in TNBC cells, we performed PI staining in MDA-MB-231 cells and quantified the percentage of cells in all cell cycle phases by flow cytometry. As shown in Figure 1B Maitake D-Fraction (IC_50_, 24 h) increased the number of cells in the subG_0_/G_1_ phase (D-Fraction = 8.92% vs vehicle = 0.96%, *p* < 0.01) and decreased those in the G_0_/G_1_ phase, compared to vehicle (D-Fraction = 48.59% vs vehicle = 75.41%, *p* < 0.001). These results suggest that Maitake D-Fraction decreases MDA-MB-231 cell viability through an induction in cell death. In order to corroborate if the increase in the number of cells in subG_0_/G_1_ phase was due to an induction of apoptosis by Maitake, AV/PI staining was performed and examined by flow cytometry after 24 h of D-Fraction or vehicle treatment. As shown in Figure 1C the apoptotic cell population (AV+) increased from 12.11% in the vehicle-treated cells to 34.31% in the D-Fraction treated cells (*p* < 0.001). Therefore, Maitake D-Fraction decreases MDA-MB-231 cell viability through induction of apoptosis.

The PI3K/AKT and MAPK/ERK are pro-survival molecular pathways that are frequently hyper-activated in many solid tumors such as breast cancer. Therefore, we examined if D-Fraction affects the activation of these signal pathways. To this end, we first evaluated the levels of Akt- and ERK1/2- phosphorylation in MDA-MB-231 cells after Maitake treatment. We found that D-Fraction attenuates Akt phosphorylation at Ser 473 (pAkt-S473) and ERK1/2 phosphorylation at Thr202/Tyr204 (PERK1/2), compared to vehicle treatment (Figure 1D). In agreement with the effect observed on pAkt-S473, we found that the levels of Bax expression remains constant but the expression of Bcl-2 decreases after D-Fraction treatment (Figure 1D). Similar results were obtained when 4T1 cells were used (data not shown). Altogether, these results suggest that D-Fraction may induce apoptosis through an attenuation of the pro-survival pathways PI3K-Akt and ERK, and through modulation of the Bax/Bcl-2 ratio.

### Maitake D-Fraction decreases the migratory capability of TNBC MDA-MB-231 and 4T1 cells

**Figure 2:**
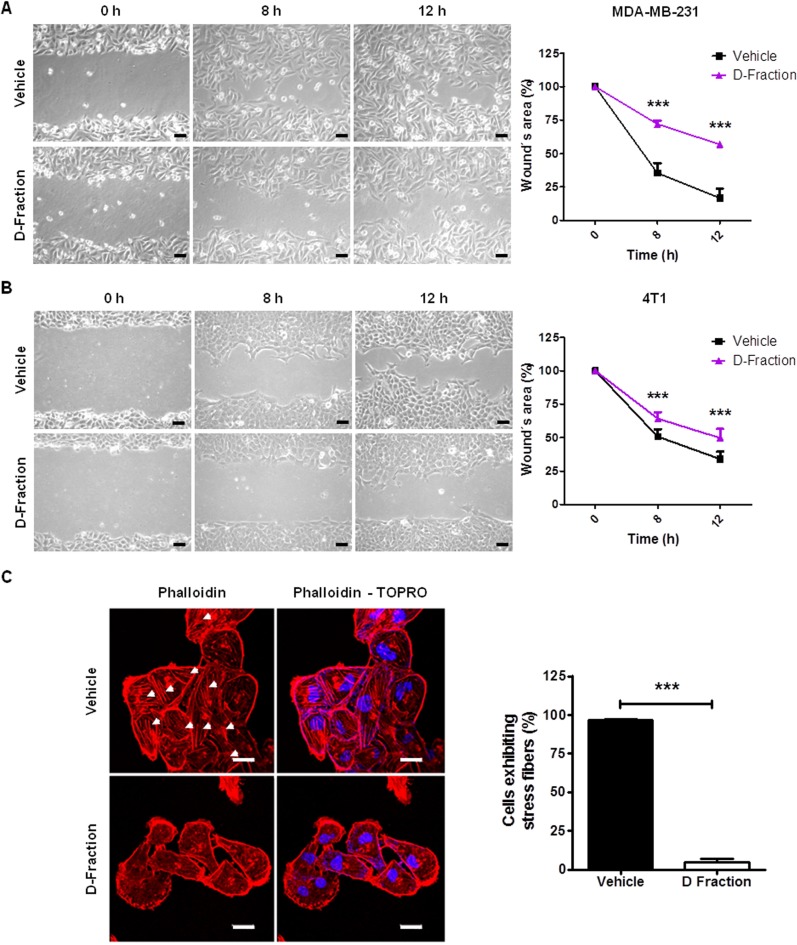
Maitake D-Fraction decreases migration of TNBC MDA-MB-231 and 4T1 cells and reduces the presence of stress fibers Representative phase-contrast pictures of the wound healing assay of (**A**) MDA-MB-231 cells and (**B**) 4T1 cells under treatment with D-Fraction (IC_50_) or vehicle. Magnification: 200×. The graphs represent the mean percentage (±SD) of uncovered wound area taking the value at 0 h as 100% of one representative experiment. Two-way ANOVA and Bonferroni post tests were performed. (**C**) Representative fluorescence images of MDA-MB-231 cells stained with rhodamine-conjugated phalloidin (F-actin) and TOPRO (nuclei) after treatment with D-Fraction (IC_50_, 12 h) or vehicle. Arrows indicate stress fibers. The graph represents the mean percentage (±SD) of cells exhibiting stress fibers after treatment of one representative experiment. Magnification: 630×, scale bars represent 20 μm. Student´s *t* test was applied. ^***^p < 0.001.

In order to determine the antimetastatic potential of Maitake D-Fraction on TNBC cells its effect on cell migration was first evaluated through wound healing assays. Confluent monolayers of MDA-MB-231 and 4T1 cells were wounded and wound closure was observed by optical microscopy during 12 h of treatment. Under treatment with D-Fraction (IC_50_), both TNBC cell lines decreased their migratory capability. Such effect was observed from 8 h of treatment onwards. At this time, uncovered wound area in D-Fraction-treated MDA-MB-231 cells was 72.04% vs 35.47% in vehicle-treated cells (*p* < 0.001, Figure 2A). In 4T1 cells, the uncovered wound area under treatment with D-Fraction was 64.37% vs 50.76% under vehicle-treatment (*p* < 0.001, Figure 2B). Neither at 8 h nor at 12 h of treatment, the cellular viability of MDA-MB-231 and 4T1 was diminished (Supplementary Figure 1).

### Maitake D-Fraction affects the reorganization of the actin cytoskeleton of TNBC MDA-MB-231 cells

Stress fibers are actin-rich structures that typically are associated with cell migration [30, 31]. Having seen the effect of Maitake D-Fraction on the migratory capability of MDA-MB-231 cells, we analyzed whether the proteoglucan alters the organization of the actin cytoskeleton of these cells by staining F-actin with rhodamine-conjugated phalloidin. As observed in Figure 2C, we found that the number of MDA-MB-231 cells with stress fibers was strongly reduced from 96.73% ± 0.31 to 4.95% ± 1.12 (*p* < 0.001) after 12 h of Maitake D- Fraction treatment (IC_50_). Furthermore, most of the D-Fraction-treated cells presented cortical actin staining. This result suggests that Maitake D-Fraction affects the organization of actin cytoskeleton reducing the presence of stress fibers, and is in concordance with the decreased migration of D-Fraction-treated MDA-MB-231 cells observed in the wound healing assay.

### Maitake D-Fraction decreases activity of MMP-2 and MMP-9 secreted by TNBC MDA-MB-231 cells

**Figure 3:**
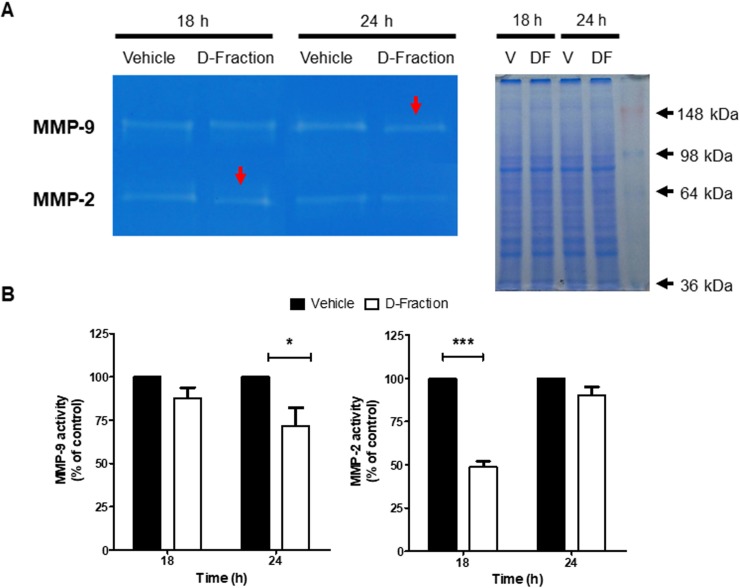
Maitake D-Fraction decreases the activity of MMP-2 and MMP-9 secreted by TNBC MDA-MB-231 cells (**A**) Representative image of gelatinolytic bands obtained with the different CM and visualized using a transilluminator (left) and its parallel coomassie gel (right). (**B**) Activity of the MMPs after D-Fraction treatment, plotted as a percentage of the control. Gelatinolytic bands were measured with Fiji (Fiji Is Just ImageJ) and normalized to total protein concentration. Values are the mean ± SEM of three independent experiments. Two-way ANOVA and Bonferroni post tests. ^*^p < 0.05, ^***^p < 0.001.

There is a well-established relationship between high activity of matrix metalloproteinases (MMPs) and tumor progression of many cancer types [32–34]. In this context, we evaluated the effect of Maitake D-Fraction on the proteolytic activity of MMP-2 and MMP-9 secreted by TNBC MDA-MB-231 cells. By gelatin zymography, we detected that D-Fraction treatment (IC_50_, 18 h) decreased MMP-2 activity by 51.06% with respect to vehicle treatment (red arrow, *p* < 0.001) (Figure 3). Furthermore, as no effect was observed in MMP-9 activity at 18 h we performed longer treatments and observed that D-Fraction (IC_50_, 24 h) decreases the activity of this secreted metalloprotease by 28.17% respect to control (red arrow, *p* < 0.05) (Figure 3). These results suggest that Maitake D-Fraction reduces the capability of TNBC MDA-MB-231 cells to degrade extracellular matrix (ECM), thereby hindering tumor invasion.

### Maitake D-Fraction promotes the intercellular adhesion and cell-substrate adhesion in TNBC MDA-MB-231 cells

**Figure 4:**
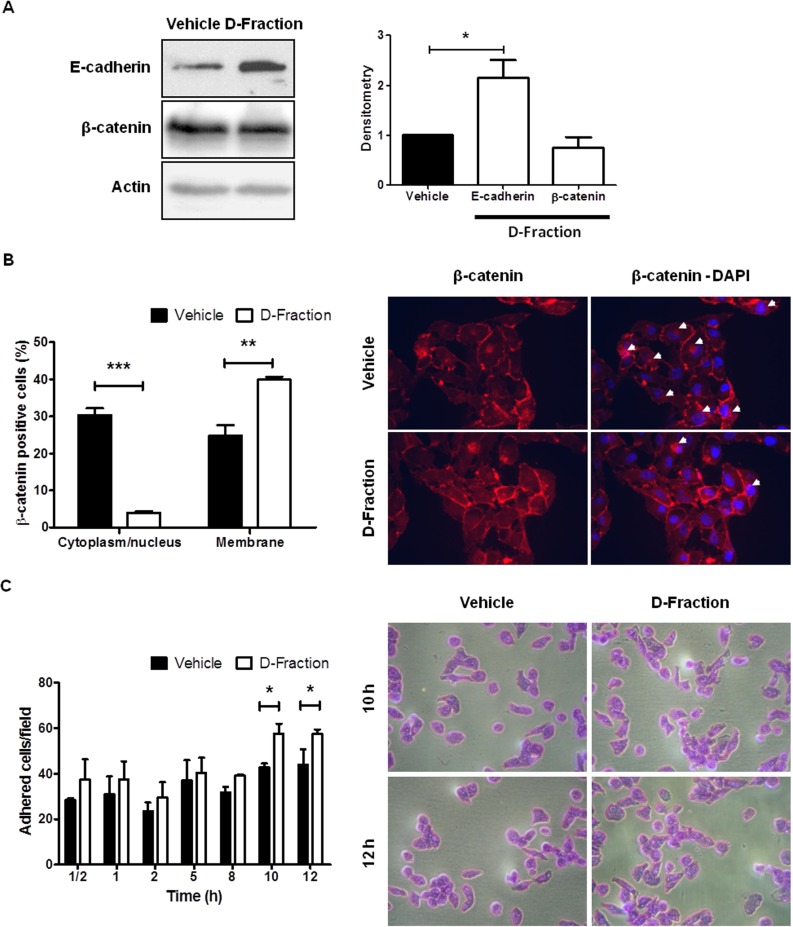
Maitake D-Fraction promotes the intercellular adhesion and cell-substrate adhesion in TNBC MDA-MB-231 cells (**A**) WB analysis for E-cadherin and β-catenin protein in cells treatment with D-Fraction (IC_50_, 12 h) or vehicle. Representative blots of at least two independent experiments and densitometry, mean ± SD. Student´s *t* test. (**B**) IF for β-catenin after treatment with D-Fraction (IC_50_, 12 h) or vehicle. The graph represents the proportion of cells (mean ± SD) with expression of β-catenin in cytoplasm/nucleus or membrane after treatment. Representative fluorescence images; arrows indicate the cytoplasm localization of β-catenin. Magnification: 400x. Student's *t* test. (**C**) Cell adhesion assay after treatment with D-Fraction (IC_50_, 12 h) or vehicle. The graph represents the number of adhered cells (mean ± SD) after treatment of one representative experiment. Representative pictures of cell adhesion assay. Magnification: 400x. Two-way ANOVA and Bonferroni post tests. ^*^p < 0.05, ^**^p < 0.01, ^***^p < 0.001.

It is well known that alterations in the adhesion properties of tumor cells play a key role in the development and progression of cancer [35, 36]. Particularly, the loss of cell-cell adhesion triggers epithelial-mesenchymal transition (EMT), a process known to contribute to the acquisition of migratory and invasive properties and favors the development of metastasis in cancer [37]. Hence, to investigate whether D-Fraction affects cell-cell adhesion we evaluated the effect of the proteoglucan on the expression of E-cadherin and β-catenin in MDA-MB-231 cells. These proteins are the major adhesion molecules at the level of the adherent junctions between epithelial cells [38, 39]. As shown in Figure 4A, we found that D-Fraction treatment (IC_50_, 12 h) increases the expression of E-cadherin in MDA-MB-231 cells compared to vehicle treatment (*p* < 0.05) but does not affect the expression of β-catenin. However, despite not changing the total cellular abundance of β-catenin, we found that Maitake D-Fraction produced a change in its subcellular localization. It has been reported that cytoplasmic and/or nuclear localization of β-catenin correlates with poor prognosis in patients with breast cancer [40–45]. As shown in Figure 4B, we found that Maitake D-Fraction decreases the presence of β-catenin in the cytoplasm/nucleus of MDA-MB-231 cells (*p* < 0.001) and promotes its membrane localization (*p* < 0.01). These results suggest that D-Fraction promotes the intercellular adhesion and the epithelial phenotype of tumor cells, thus decreasing their invasive potential.

On the other hand, we evaluated if Maitake D-Fraction affects the adhesion of MDA-MB-231 cells to the substrate. With this end, MDA-MB-231 cells were pre-treated with D-Fraction (IC_50_, 12 h) or vehicle. Then, the cells were harvested and incubated for different times in 96-well plates. As shown in Figure 4C, after incubating cells for 10 h (vehicle 42.83 vs D-Fraction 57.60 adhered cells) and 12 h (vehicle 44.03 vs D-Fraction 57.43 adhered cells), D-Fraction significantly increased MDA-MB-231 cell adhesion compared to vehicle (*p* < 0.05). Therefore, Maitake D-Fraction is capable of increasing the adhesive response of TNBC MDA-MB-231 cells, suggesting that D-Fraction induces a less malignant phenotype.

### Maitake D-Fraction decreases the invasive capability of TNBC MDA-MB-231 and 4T1 cells

**Figure 5:**
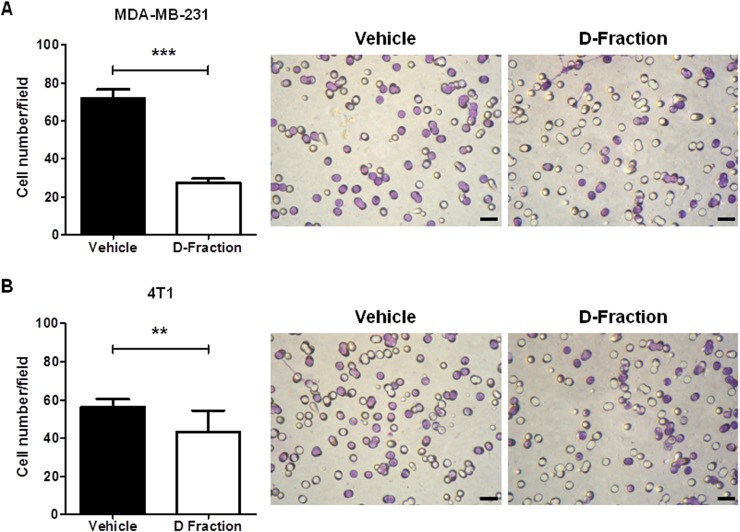
Maitake D-Fraction decreases invasion of TNBC MDA-MB-231 and 4T1 cells The graph represents the mean (± SEM) number of (**A**) MDA-MB-231 and (**B**) 4T1 invasive cells after treatment with D-Fraction (IC_50_, 12 h) or vehicle. The assays were performed in triplicate. Student's *t* test was applied; ^**^p < 0.01, ^***^p < 0.001. Representative pictures of cell invasion assays for each TNBC cell line. Magnification: 400×.

The metastatic process begins with local tumor invasion into surrounding tissue, in which factors such as cell adhesion to the substrate, degradation of ECM and tumor cell motility play a key role [46–48]. Since the results of the previous sections show that Maitake D-Fraction affects these factors tending to decrease the metastatic potential of TNBC MDA-MB-231 cells, we decided to investigate the overall effect of D-Fraction on the invasive process. To do this, we evaluated the invasive capacity of tumor cells using transwell chambers (Millipore) with Matrigel. As shown in Figure 5A Maitake D-Fraction treatment reduced the invasiveness of MDA-MB-231 when compared with vehicle treatment (mean value of vehicle treatment = 71.96 vs D-Fraction treatment = 27.54, *p* < 0.001). In addition, we also found that D-Fraction decreases the invasive capability of 4T1 cells (mean value of vehicle treatment = 56.28 vs D-fraction treatment = 43.38, *p* < 0.01; Figure 5B).

### Maitake D-Fraction retards tumor growth and reduces the number of lung metastases in a xenograft murine model of TNBC human cells

**Figure 6:**
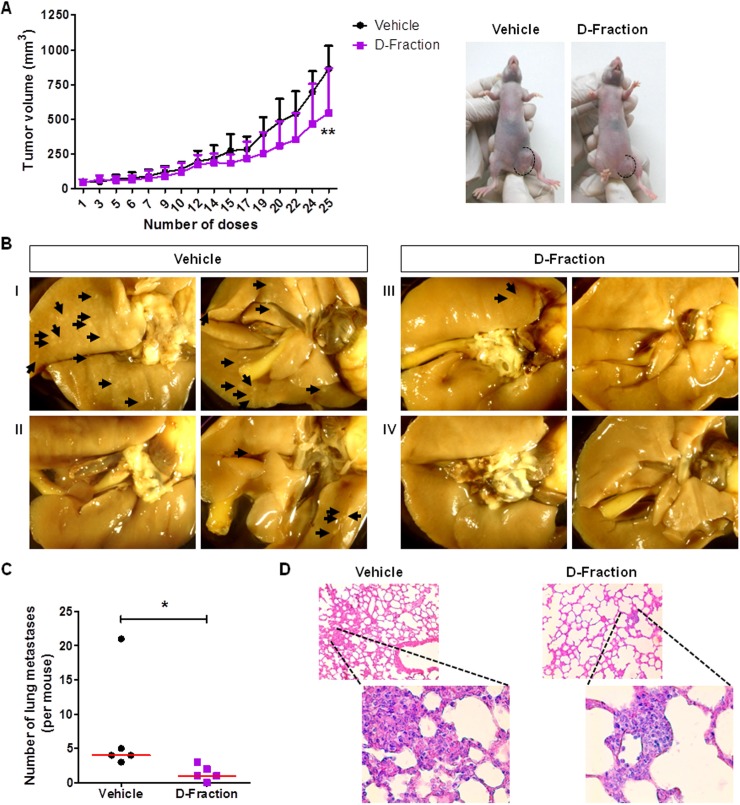
Maitake D-Fraction retards tumor growth and reduces the number of lung metastases in a xenograft model of TNBC human cells Tumor-bearing nude mice were treated with D-Fraction (21.87 mg × Kg^-1^ × day^–1^; *n* = 5) or vehicle (*n* = 5). (**A**) Graph showing the kinetic of tumor growth and representative images of tumor-bearing mice treated with D-Fraction or vehicle at the end of the treatment (on the right). Values are the mean ± SD. Two-way ANOVA and Bonferroni post test were used. (**B**) Representative pictures of the lung metastases from mice treated with vehicle (I and II) and D-Fraction (III and IV). Shown are the two sides of lungs. (**C**) Graph showing the quantification of the number of lung metastases per mouse. The red lines represent the median value. Mann-Whitney *U* test was performed. (**D**) Representative pictures of the metastases observed in H&E-stained lung slides from mice treated with vehicle and D-Fraction. Magnification: 100× and 400×, for the amplifications. ^*^p < 0.05, ^**^p < 0.01.

Having demonstrated that Maitake D-Fraction decreases the viability and the metastatic potential of TNBC MDA-MB-231 and 4T1 cells grown *in culture*, we aimed to evaluate the antitumor and anti-metastatic activity of D-Fraction *in vivo*. Therefore, we employed an orthotopic model of human breast cancer MDA-MB-231 cells. This model allows to evaluate both the primary tumor growth and the development of lung metastases [49]. We found that Maitake D-Fraction retarded tumor growth when compared to the vehicle-treated mice (Figure 6A). Indeed, D-Fraction-treated mice presented smaller tumors than vehicle-treated mice (544.1 mm^3^ vs. 862.7 mm^3^; *p* < 0.01). In addition, the lungs fixed in Bouin´s solution were examined. As shown in Figure 6B, small superficial metastases were detected and quantified. We found a lower number of lung metastases per animal in D-Fraction-treated mice (median = 1, range 0 – 3) than in the vehicle-treated mice (median = 4, range 3 – 21) (*p* < 0.05; Figure 6C). Finally, the presence of metastases were confirmed by staining with Hematoxylin and Eosin (H&E) (Figure 6D). Altogether, these *in vivo* results demonstrate the antitumoral and antimetastatic activity of D-Fraction in a xenograft murine model of TNBC, and are in agreement with the *in culture* effects of D-Fraction on TNBC cells.

## DISCUSSION

Breast cancer, the most common cancer in women worldwide, is a heterogeneous disease, composed of a broad spectrum of tumor subtypes with different biological characteristics and clinical behavior [6]. The primary breast cancer markers traditionally used in routine clinical practice to establish the diagnosis, prognosis and therapy of disease are ER, PR and HER2 [50]. Thus, tumors that express ER and PR are treated with agents that interfere with hormone production or action, plus chemotherapy. Tumors with over-expression and/or gene amplification of HER2 are treated with agents that inhibit HER2, plus chemotherapy [8]. On the contrary, there is no targeted therapy for tumors that do not express any of three markers (triple negative) [51] so they can only be treated with chemotherapy [8]. Therefore, new effective and safe drugs for the treatment of highly aggressive TNBC are urgently needed to be found.

In addition to the excellent nutritional value, medicinal aspects of mushrooms extracts or their metabolites make them excellent candidates for the search and development of new drugs [11, 23, 52]. In this field, many mushroom species are valued as small pharmaceutical factories producing hundreds of bioactive compounds, many of which have shown to exert therapeutic action against the development of cancer [10, 11]. Such is the case of D-Fraction, a bioactive proteoglucan extracted from *Grifola frondosa* mushroom, which in turn is a dietary supplement.

In general, mushroom metabolites with antitumor activity exert their effect exclusively by activation of the immune response of the host organism or acting directly on the tumor cell, interfering with particular cellular signal transduction pathways linked to cancer development and progression [53–55]. Unlike these, Maitake D-Fraction, attacks tumor cells both by their immunostimulatory capacity [14–21] and independently of the immune system [22–29]. Particularly with respect to the immuno-independent antitumor effect of D-Fraction, it has been observed to be tumor-type specific.

In this study, we demonstrated for the first time that Maitake D-Fraction is able to act directly on TNBC MDA-MB-231 and 4T1 cells, by modulating different cellular capabilities whose deregulation lead to the development and progression of cancer and thus decreasing the highly aggressive phenotype of these tumor cells.

The present data show that Maitake D-Fraction decreases the viability of TNBC MDA-MB-231 cells through apoptosis induction. In our previous works we have demonstrated that D-Fraction also decreases the viability of hormone-dependent MCF7 [24] and hormone-independent LM3 breast cancer cells [25]. These results indicate that Maitake D-Fraction affects breast cancer cell viability regardless of hormone receptors and HER2 status of tumor cells. This capability exerted by D-Fraction is not a common feature of medicinal mushrooms extracts. Of 38 extracts evaluated on MCF7 (hormone-dependent), BT-20 and MDA-MB-231 (TNBC) cells, only three of them were able to affect the cell viability of both tumor subtypes [52]. In general, mushrooms extracts only decrease the viability of ER positive breast cancer cells by inhibiting aromatase activity [53, 54, 56, 57]. In this way, Maitake D-Fraction is a potential agent with broad-spectrum antitumor activity against the different breast tumor subtypes: hormone-dependent, hormone-independent and triple-negative.

One of the main limitations in cancer treatment is the lack of therapies that can efficiently prevent the development of metastasis. Metastatic breast cancer is essentially incurable and is responsible for most of breast cancer deaths. It is known that TNBC patients have a higher risk of metastatic disease, compared to patients with other breast cancer subtypes [6]. Moreover, patients with metastatic TNBC have a shorter survival time [58]. Despite the importance of the metastatic process, therapeutic approaches of mushrooms extracts against the “metastatic phenotype” are far behind in terms of development when compared to the mushroom extract effects against other “hallmarks of cancer” such as sustained proliferation and evasion of apoptosis. Therefore, in the present work, in addition to demonstrating that proteoglucan decreases the viability of TNBC MDA-MB-231 cells, we also evaluated its effect on the metastatic potential of these tumor cells. We found that D-Fraction affects different abilities employed by tumor cells in the different stages of the metastatic process.

On the one hand, Maitake D-Fraction significantly increased E-cadherin expression in TNBC MDA-MB-231. E-cadherin is one of the main cell adhesion molecules between epithelial cells [38, 39] and its loss of expression is considered a hallmark in the EMT [37]. It is well known that EMT is a key process in the progression of epithelial cancer by which tumor cell loses cell-cell adhesion, detaches itself from the primary tumor and assumes a mesenchymal-like phenotype to migrate and invade the surrounding tissue [30, 37]. Furthermore, it is well known that the cytoplasmic domains of E-cadherin interact with β-catenin, and this reciprocal action is crucial for the adhesive function of E-cadherin [40]. Under pathologic conditions such as cancer, the E-cadherin/β-catenin complex is generally destroyed, leading to impairment of cell-cell adhesion [40]. Upon loss of its membrane location, free β-catenin accumulates in the cytoplasm and/or translocates to the nucleus. In patients with breast cancer, the cytoplasmic and/or nuclear localization of β-catenin has been correlated with poor prognosis [40–45]. In this work, Maitake D-Fraction decreased the presence of β-catenin in the cytoplasm/nucleus of MDA-MB-231 cells and promoted its membrane localization suggesting its interaction with E-cadherin. Altogether, our result suggests that D-Fraction decreases the invasive potential of TNBC MDA-MB-231 cells by favoring intercellular adhesion and promoting its epithelial phenotype.

The present data also show that Maitake D-Fraction decreases the migratory capability of TNBC MDA-MB-231 cells. On the one hand, we demonstrated that proteoglucan affects the actin cytoskeleton re-organization, which is an essential mechanism of cellular motility for most types of cell migration [59]. Filopodia, lamellipodia and stress fibers are different kinds of actin structures, which generate the intracellular forces necessary to cell translocation [30, 31]. Particularly, D-Fraction decreases the motility of the MDA-MB-231 cells by reducing the presence of stress fibers in them. In concordance with this, MDA-MB-231 cells have been classified as mesenchymal-like cell line [60] and a characteristic of mesenchymal-migrating cells is the presence of actin stress fibers [30, 37]. On the other hand, we demonstrated that Maitake D-Fraction is able to specifically affect the mesenchymal migration mode, which is adopted by tumor cells after the acquisition of mesenchymal phenotype through EMT [61]. This type of motility is dependent on the use of proteases to degrade the surrounding ECM and generate a path through which cells can invade [62, 63]. There is a strong correlation between the expression, secretion and/or activation of different proteases with the tumor ability to metastasize [33]. In this work, Maitake D-Fraction was shown to decrease the activity of MMP-2 and MMP-9 secreted by TNBC MDA-MB-231 cells, two MMPs clearly linked to breast cancer metastatic potential [64, 65]. In this context, D-Fraction could be employed as an inhibitor of MMPs activity to reduce the invasive potential of TNBC.

Unfortunately, cancer cells may maintain migratory capacity after the abolition of their ability to degrade ECM [63, 66]. It has been shown that in the presence of proteolytic inhibitors, MDA-MB-231 cells spontaneously switch from proteolytic mesenchymal-like migration to protease independent amoeboid-like migration, in order to continue migrating [67]. This cellular plasticity makes it difficult to repress cancer cell invasion by using only protease inhibitors. However, another fundamental requirement for switching to an amoeboid migration is the loss of cell-substrate adhesion [59]. In this work, we demonstrated that Maitake D-Fraction increases the adhesion of MDA-MB-231 cells to substrate, thereby decreasing their metastatic ability. Therefore, this result suggests that the proteoglucan could limit the ability of TNBC cells to adopt protease-independent amoeboid-like migration. In concordance with our result, [68] and [69] have reported that decreases in cell-substrate adhesion in LMM3 (murine breast cancer line) and MDA-MB-231 respectively, is associated with an increase in their migratory and metastatic potential.

In concordance with all the effects of Maitake D-Fraction tending to decrease invasive ability of MDA-MB-231 cells, the invasion assay with Matrigel demonstrated that proteoglucan effectively decreases invasive capacity of these highly aggressive TNBC cells.

Finally, in a xenograft model of TNBC human cells we demonstrated that Maitake D-Fraction treatment retards tumor growth and reduces the number of spontaneous lung metastases. These *in vivo* effects corroborate the *in culture* effects of D-Fraction that we demonstrated in MDA-MB-231 cells.

In conclusion, we demonstrate that Maitake D-Fraction decreases the *viability* of TNBC MDA-MB-231 cells through *apoptosis* induction and affects diverse abilities employed by tumor cells in the different stages of the metastatic process, thus decreasing the highly aggressive phenotype of these tumor cells. D-Fraction increases the *adhesion* of MDA-MB-231 cells between them, by increasing the expression of E-cadherin and promoting the membrane localization of β-catenin, and increasing the adhesion of MDA-MB-231 to the substrate. Also, the proteoglucan decreases the *motility* of MDA-MB-231 cells by affecting the actin cytoskeleton rearrangements. Furthermore, D-Fraction decreases the *proteolytic activity* of MMP-2 and MMP-9 secreted by MDA-MB-231 cells. The coupling of all these effects led to a decrease in the *invasive* capacity of MDA-MB-231 cells after D-Fraction treatment. In concordance with these results, we also demonstrate that Maitake D-Fraction treatment retards tumor growth and reduces the number of lung metastases in a xenograft model of TNBC human cells. Therefore, these results widen the antitumoral spectrum of D-Fraction within the heterogeneity of breast cancer and allow us to infer that Maitake D-Fraction may serve as a novel natural drug against TNBC.

## MATERIALS AND METHODS

### Bioactive maitake D-Fraction

D-Fraction is a proteoglucan extracted from the medicinal mushroom *Grifola frondosa* (Maitake). In this work we use Maitake D-Fraction^®^ Pro 4X Liquid, which is made from D-Fraction extract, vegetable glycerin and water by Mushroom Wisdom, Inc. (formerly Maitake Products, Inc.) New Jersey, USA. Maitake D-Fraction^®^ Pro 4X Liquid was prepared by a standardized procedure developed by Mushroom Wisdom, Inc. and does not contain alcohol, sugar, yeast, mold, corn, salt, wheat, artificial color, preservatives or synthetic pesticides or fertilizers.

### Cell culture

MDA-MB-231 human and 4T1 murine TNBC cell lines were employed and were a generous gift from Instituto de Oncología Ángel Roffo and UBA-IFIBYNE-CONICET, respectively. The cells were cultured in RPMI (Sigma) supplemented with 10% (v/v) fetal bovine serum (FBS, Gibco), L-glutamine (5 mM, Gibco), penicillin (100 U/mL, Gibco) and streptomycin (100 μg/mL, Gibco) at 37° C in a humidified 5% CO_2_ air atmosphere.

### Cell viability assays

The MDA-MB-231 and 4T1 cells were plated at a density of 3500–3000 cells/well respectively into 96 multi-well dishes in complete medium. They were treated with 30, 300, 750, 1500 and 2250 μg/mL of D-Fraction or vehicle for 24, 48 and 72 h. Then, they were counted manually using a hemocytometer, as previously described [70]. The experiment was repeated at least twice and performed in quadruplicate. Half maximal inhibitory concentrations (IC_50_) of D-Fraction were calculated using the sigmoidal dose-response equation employing GraphPad Prism 5.00 program. Additionally, cell viability was assessed by 4-[3-(4-Iodophenyl)-2-(4-nitrophenyl)-2H-5-tetrazolio]-1,3 benzene disulfonate (WST-1) colorimetric assay (Roche) as previously described [25]. Vehicle treatment did not exert effects over cellular viability when compared to non-treated cells.

### Cell cycle analysis

Cell cycle analysis was performed as previously described [71]. In brief, MDA-MB-231 cells were treated with D-Fraction (IC_50_, 24 h) or vehicle. After that, they were stained with PI (Roche), and analyzed for DNA content by FACScan Calibur Becton Dickinson. Data were analyzed by CellQuest software (Becton Dickinson). At least 100,000 cells were analyzed for each sample.

### Apoptosis analysis

Apoptosis was analyzed by flow cytometry using Annexin V-FITC (AV)/PI double staining as previously described [70]. In brief, MDA-MB-231 cells were treated with D-Fraction (IC_50_, 24 h) or vehicle. The cells were counted using FACScan Calibur Becton Dickinson and data were analyzed by CellQuest software (Becton Dickinson). We determined the total apoptotic population including both early apoptotic cells (AV+/IP-) and late apoptotic cells (AV+/IP+). At least 100,000 cells were analyzed for each sample.

### Cell migration assay

Cell migration was studied by employing the wound healing assay. Briefly, the cells were seeded in 35-mm Petri dishes and cultured until confluence. MDA-MB-231 and 4T1 cells were treated with D-Fraction (IC_50_) or vehicle. The cells were scraped with a 200 μL micropipette tip, denuding a strip of the monolayer. Then, they were observed and photographed every 4 h and for a shorter time than the doubling time (T_2_) of the cell line (MDA-MB-231 T_2_ = 29 h; 4T1 T_2_ = 15.5 h). Images were captured with an inverted microscope (Nikon Eclipse TE2000-S) equipped with a digital camera (Nikon Coolpix S4). The uncovered wound area was measured and quantified at different intervals with Fiji (Fiji Is Just ImageJ).

### Analysis of actin cytoskeleton

The actin cytoskeleton was visualized by staining with rhodamine-phalloidin as previously described [25]. Briefly, MDA-MB-231 cells were seeded on glass coverslips in 35-mm Petri dishes and cultured until 50% confluence. They were treated with D-Fraction (IC_50_, 12 h) or vehicle. After treatment, they were incubated with rhodamine-conjugated phalloidin (1:100) for 30 min and with TOPRO (1:1000) for 5 min. Fluorescence images were acquired with a confocal microscope (Leica TCS SP2). For each replicate, at least 400 cells in 400x random fields were evaluated and the proportion of stress fiber-containing cells was determined.

### Immunofluorescence (IF)

IF was performed as previously described [70]. Briefly, MDA-MB-231 cells were seeded on glass coverslips in 35-mm Petri dishes and cultured until 50% confluence. They were treated with D-Fraction (IC_50_, 12 h) or vehicle. After that, the cells were incubated with the primary antibody rabbit polyclonal anti-β-catenin (H-102) (Santa Cruz Biotechnology, sc-7199) for 1 h and then incubated with Alexa Fluor^®^ 568 goat anti-rabbit IgG (Molecular Probes, Invitrogen) for 1 h in the dark. Finally they were stained with 4’,6-diamidino-2-phenylindole (DAPI; 1:10000). Fluorescence images were acquired with a Nikon Eclipse E-600 microscope (Nikon, Melville, NY, USA), equipped with a SBIG model ST-7 camera (Santa Barbara Instrument Group). For each replicate, at least 400 cells in 400× random fields were evaluated and the proportion of cells expressing β-catenin in membrane or cytoplasm/nucleus was determined.

### Preparation of conditioned media (CM)

Secreted MMP activity was evaluated in CM. Briefly, semi-confluent cell monolayers growing in 100-mm Petri dishes were treated with D-Fraction (IC_50_) or vehicle for 6 and 12 h. Then, the cells were incubated overnight with 4 mL of serum-free RPMI in presence of D-Fraction (IC_50_) or vehicle. CM were individually collected, centrifuged (1200 rpm for 3 min, in cold), aliquoted and stored at –20° C. The remaining monolayers were lysed using lysis buffer with protease inhibitors (Calbiochem) and cell protein content was determined (Bradford Reagent, Bio-Rad).

### Zymography for MMP-2 and MMP-9

MMP-2 and -9 activities in CM from MDA-MB-231 cells were studied by measuring collagenolytic activity in substrate-impregnated gels, as previously described [25]. Activity bands were visualized by negative staining. Gelatinolytic bands were visualized using a transilluminator and measured with Fiji (Fiji Is Just ImageJ). Data were normalized to total protein concentration.

### Cell adhesion assay

Cell adhesion was evaluated as previously described [72], with some modifications. Briefly, MDA-MB-231 cells were treated with D-Fraction (IC_50_, 12 h) or vehicle. Then, cells were seeded into 96-well culture plates (10000 cells/well; 3 wells/condition) and were allowed to adhere. The viability of the seeded cells was verified using Trypan Blue (Sigma) exclusion assay. After 30 min, 1 h, 2 h, 5 h, 8 h, 10 h and 12 h, non-adherent cells were removed by gentle washing and attached cells were stained with 0.1% (w/v) Crystal Violet (Sigma) for 20 min. Cells attached to the culture plate were observed with an inverted microscope (Nikon Eclipse TE2000-S), equipped with a digital camera (Nikon Coolpix S4). For each replicate, ten randomly selected fields were photographed and the cells were counted with Fiji (Fiji Is Just ImageJ).

### Western blot

The MDA-MB-231 cells were plated into dishes containing complete medium and treated with D-Fraction (IC_50_) or vehicle for 15 min, 60 min, 12 h or 18 h. The treatment time varies according to the protein under study and, particularly for evaluating the expression of phospho-Akt-S473 (pAkt-S473) and phospho-ERK1/2 (pERK1/2), cells were previously deprived of FBS for 18 h. Then, they were lysed and the total protein concentrations were determined as previously described [25]. Lysates were prepared to examine the expression of pAkt-S473, total Akt (Akt), pERK1/2, total ERK1/2 (ERK1/2), Bax, Bcl-2, E-cadherin and β-catenin. Primary antibodies used were rabbit polyclonal anti-phospho-Akt (Ser 473) (Cell Signaling, #9271S), rabbit polyclonal anti-pERK1/2 (Cell Signaling, #9101), rabbit polyclonal anti-Bax (N-20) (Santa Cruz Biotechnology, sc-493), mouse monoclonal anti-Bcl-2 (C-2) (Santa Cruz Biotechnology, sc-7382), rabbit polyclonal anti-E-cadherin (H-108) (Santa Cruz Biotechnology, sc-7870) and rabbit polyclonal anti-β-catenin (H-102) (Santa Cruz Biotechnology, sc-7199). Rabbit polyclonal anti-Akt (Cell Signaling, #9272) and mouse monoclonal anti-ERK1/2 (C-9) (Santa Cruz Biotechnology, sc-514302) were used for normalization of Akt and ERK1/2 phosphorylation. Anti-β-actin (C-11, polyclonal goat, Santa Cruz Biotechnologies, sc-1615) was used as internal control for protein loading and analysis.

### Cell invasion assay

The invasion of cancer cells was assessed in transwell chambers (Millipore) with Matrigel (BD Bioscieces) as previously described [70]. In brief, each transwell was coated with 100 μL of a 1:3 matrigel in cold serum-free RPMI. The lower chamber was filled with 0.6 mL of complete RPMI medium. MDA-MB-231 or 4T1 cells (12500 cells/well) and D-Fraction (IC_50_) or vehicle were suspended in complete RPMI medium (0.4 mL/well), placed in the upper transwell chambers in triplicates, and incubated for 12 h at 37° C. After incubation, the invading cells on the lower side of the membrane were stained with 0.5% (w/v) Crystal Violet (Sigma) for 5 min. For each replicate, 10 randomly selected fields were photographed and the cells were counted with Fiji (Fiji Is Just ImageJ).

### Xenograft murine model of human breast cancer

*In vivo* studies were conducted in accordance with the National Institutes of Health (NIH) Guide for the Care and Use of Laboratory Animals. Six-week-old virgin female N:NIH(S)-Fox1^nu^ mice, weighing around 24 g, were purchased from Facultad de Ciencias Veterinarias (La Plata, Argentina). Animals were given free access to water and food, and were housed in a climate-controlled room with a 12-h light/12-h dark cycle. Ten animals were injected with MDA-MB-231 cells as previously described [73], with some modifications. In brief, 4.8 × 10^6^ MDA-MB-231 cells suspended in 75 μL of serum-antibiotic-free RPMI and 75 μL of Matrigel (BD Bioscieces) were implanted subcutaneously in the mammary fat pad of the nude mice. The cells were implanted using cold 1-mL syringes with needles (BD) 0.80 × 25 mm (21Gx1). When tumors reached a width > 30 mm, MDA-MB-231 tumor-bearing mice were randomly divided into two groups and were injected as indicated previously [25] :1) 5 mice with D-Fraction (21.87 mg/Kg in 50 μL of saline) and 2) 5 mice with vehicle of D-Fraction (glycerin-water in 50 μL of saline). Mice were injected subcutaneously in the tumor periphery five times a week for five weeks (total doses = 25). Tumor growth was measured each other day with digital calipers and tumor volume was calculated as π/6 × a × b^2^, where a is the length and b is the width in millimeters. At the end-point animals were sacrificed by cervical dislocation. Lungs were removed and fixed in Bouin's solution. The superficial lung metastases per mouse were counted by two investigators with the aid of a Stereo Microscope (Nikon SM Z1500) coupled with High-Intensity Illuminator (Nikon NI-150) and a digital camera (Nikon DXM 1200F). After that, the lungs were processed into paraffin by standard procedures, five-micrometer sections were obtained and stained with H&E for histological confirmation of the metastasis.

### Statistical analysis

The GraphPad Prism software package, version 5.00 was used for collection, processing and statistical analysis of all data. Cell viability, MMP-s activity, adhesion, migration, cell cycle assays, relative Akt and ERK1/2 phosphorylation and *in vivo* comparison of the tumor volume were analyzed with two-way Analysis Of Variance (ANOVA) and Bonferroni post tests. Apoptosis assay, IF, WB of Bax, Bcl-2, E-cadherin and β-catenin, cell invasion assay and effects on actin cytoskeleton were analyzed with Student's *t* test. Comparison of the number of lung metastases among different groups was analyzed by the non-parametrical Mann–Whitney *U* test. Statistical significance was determined at *p* < 0.05 level.

## SUPPLEMENTARY MATERIALS FIGURES



## Abbreviations

ANOVA: analysis of variance
AV: Annexin V-FITC
CM: conditioned media
ECM: extracellular matrix
EMT: epithelial-mesenchymal transition
ER: estrogen receptor
FBS: fetal bovine serum
HER2: human epidermal growth factor receptor 2
H&E: Hematoxylin and Eosin
IC_50_: half maximal inhibitory concentrations
IF: immunofluorescence
MMP: matrix metalloproteinase
PI: propidium iodide
PR: progesterone receptor
T_2_: doubling time
TNBC: triple-negative breast cancer
WB: western blot
WST-1: 4-[3-(4-Iodophenyl)-2-(4-nitrophenyl)-2H-5-tetrazolio]-1,3 benzene disulfonate
