# Supplementary material for: Antitumoral and antimetastatic activity of Maitake D-Fraction in triple-negative breast cancer cells
Source: Oncotarget. 2018 May 4;9(34):23396–412. doi: 10.18632/oncotarget.25174 (PMC5955106; doi:10.18632/oncotarget.25174)
Supplement: Supplementary file 1 [file oncotarget-09-23396-s001.pdf]

Antitumoral and antimetastatic activity of Maitake D-Fraction in triple-negative breast cancer cells

SUPPLEMENTARY MATERIALS

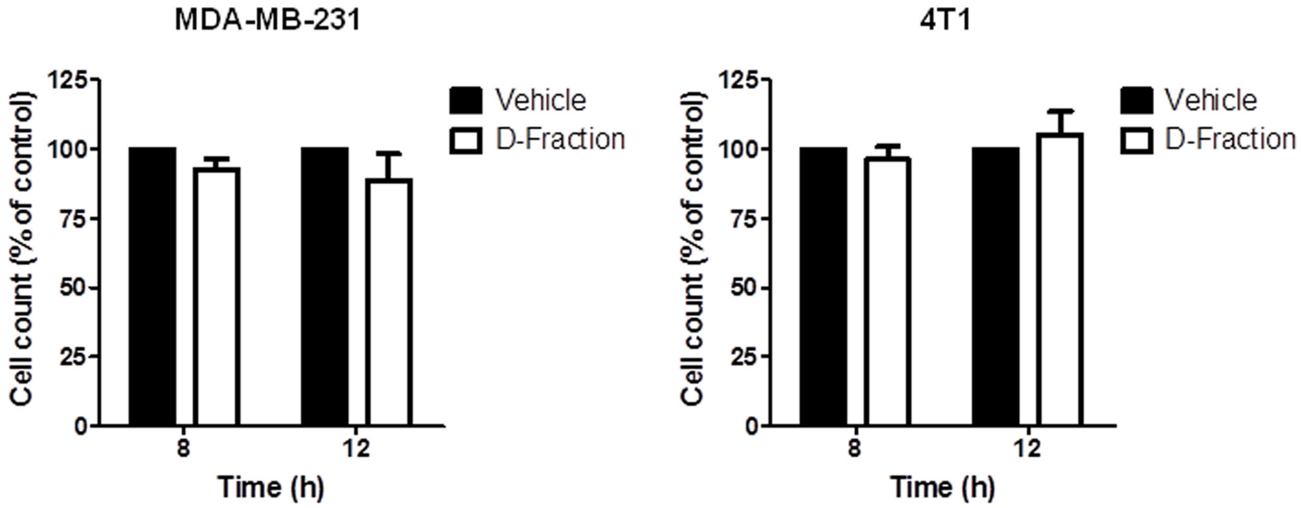

Supplementary Figure 1: Maitake D-Fraction does not affect the viability of TNBC cells at treatment times that decrease their migration. Cell count was assessed in MDA-MB-231 and 4T1 cells after 8 or 12 h of treatment with D-Fraction (IC50) or vehicle. Data show the percentage of cells in relation to vehicle-treated cells. The bars represent the mean ± SEM of two independent experiments.
